# Editorial: Education and Training in Biomedical Science

**DOI:** 10.3389/bjbs.2024.13598

**Published:** 2024-09-02

**Authors:** Sheri Scott, Beverley Cherie Millar, Stephen McClean

**Affiliations:** ^1^ School of Science and Technology, Nottingham Trent University, Nottingham, United Kingdom; ^2^ School of Biomedical Sciences, Ulster University, Coleraine, United Kingdom; ^3^ Northern Ireland Public Health Laboratory, Belfast City Hospital, Belfast, United Kingdom

**Keywords:** biomedical science, continual professional development, education, employability, laboratory medicine, Training, pedagogy

## Introduction

Biomedical science is an expanding discipline encompassing healthcare delivery, technological advances, and scientific research. Whether a biomedical science graduate is entering a regulated healthcare profession or uses their education as a platform for further study, leading to varied careers both within and outside of healthcare, it is important that biomedical science education providers deliver high quality educational and training programmes, which provide opportunities to develop essential skills required for tomorrow’s workforce.

Higher qualifications in biomedical science offer a gateway to a diverse range of career pathways and ongoing professional development. Whether employment is sought in a clinical laboratory environment in a healthcare related discipline (medical microbiology, clinical chemistry, haematology, transfusion, cell pathology, immunology and genetics), research and development, teaching, communication and bioinformatics or careers encompassing environmental, pharmaceutical, nutrition and forensic sectors, the skills and knowledge required are a key concern in curriculum development.

This Special Issue “*Education and Training in Biomedical Science*” showcases best practices in pedagogical approaches which have recently impacted upon teaching, workplace training and assessment to ensure graduates have the knowledge and skills required for employment within the biomedical science sector. This editorial provides context and a snapshot glance of these approaches.

## Course Delivery

The last 4 years have seen a transformation in the way biomedical science education is delivered and the acquired knowledge assessed. The COVID-19 pandemic brought unprecedented and unplanned changes in educational delivery during 2020–2021. Rapid moves to online delivery, utilisation of digital pedagogies and adopting virtual assessments became a necessity in order for students to complete higher education programs (Pearse and Scott). Online and blended-learning approaches have incrementally increased in popularity [1–3]. McKenna’s paper discusses the provision of “dry-lab” final year honours projects, as a viable alternative to traditional “wet-lab” projects. The main themes of the study encompassed expectations, skills and employability, quality of experience and choice. The findings support the rationale for dry projects as a suitable and equitable alternative for wet-lab project provision.

Online learning offers flexibility in study and a more environmentally sustainable education option. The ongoing development of digital technologies, support a wide variety of undergraduate and post graduate level programmes. Despite the popularity of such programmes, online learning provides minimal opportunities for face-to-face interaction, subsequently impacting on student programme satisfaction, engagement and peer communication. Millar et al. explore the pedogeological approach of how group assessments can help build online learning communities in Biomedical Science Distance Learning Programmes. Student reflections provided the basis for the evaluation. Enjoyment, collegiality, the development of digital skills and the gaining of knowledge scored the highest in the student reflections.

## Curriculum Developments

Recent changes in HCPC standards of Proficiency (SoPs) [4] and the Biomedical Science QAA benchmark statement [5] have further initiated changes in Biomedical Science curriculums. Science communication, quality assurance, equality, diversity and inclusion, point-of-care-testing (POCT) and sustainability are now required as part of curriculum enhancements. In a second paper by Millar et al., curriculum inclusion of scientific communication and digital capabilities is presented. In this paper, details on the production of a co-designed online scientific communication and digital capabilities resource is provided, whereby students gain creative, digital, analytical and scientific communication skills including lay writing and visual abstracts, enabling students to communicate with individuals with different levels of understanding using different formats aligning with the HCPC SoPs and QAA benchmark [4, 5]. The findings of this research supported development of transferable skills applicable to student future career choices.

The recent HCPC SoPs changes and the HCPC Standards of Education and training in emphasise the need for interprofessional learning opportunities and service user involvement in applied biomedical science undergraduate courses [4, 6]. Students are expected to demonstrate the ability to build and sustain professional relationships and participate in training that supports high standards of practice, professional conduct and positive interpersonal relationships. Two papers by Bashir et al., look at service user involvement (Bashir et al.) and interprofessional learning, respectively (Bashir et al.). Bashir et al. first presents a workshop where patients discuss how pathology services have contributed to their medical care, while service providers discuss their roles and their interactions with the pathology services. Outcomes from the workshop include the reinforcement of “a patient behind each sample” and the incorporated student reflection highlighted potential improvements to pathology services. Bashir et al.’s second paper looked at using a cytomegalovirus (CMV) case study with Audiology and Biomedical Science Students. Over 82.4% of respondents either “agreed” or “strongly agreed” that understanding of the roles of other healthcare professions is needed for successful career development.

A key component of clinical modules taught on IBMS accredited and HCPC approved Biomedical Science undergraduate programmes, is the fundamental requirement for students to be able to apply theory to practice, significantly in the form of clinical case interpretation and the diagnosis of patients from presented results [7]. Posner et al., present a problem-based learning approach to case study interpretation. The aim was to improve engagement, skill acquisition and the student experience by utilising active student-centred methods, to improve student self-learning. Results from the study demonstrated improved student engagement and an improved student experience. Similarly, Bashir et al. measured the impact of incorporating case study presentations into applied biomedical science placement workshops for Trainee Biomedical Scientists. In this paper, the study aimed to evaluate the effectiveness of a redesigned workshop where students generated and presented medical case studies to peers, academics, and training leads. Findings from the study not only showcased a unique collaborative partnership between higher education institutions and pathology laboratories but evidenced enhanced student confidence in 1) the knowledge of clinical conditions, 2) presentation skills, and 3) ability to think critically.

## Competencies and Employability

Higher education providers providing IBMS accredited and HCPC approved programmes, strive to produce high quality graduates attractive to employers. These graduates in biomedical science need both discipline specific knowledge and skills, plus transferable skills essential for HCPC regulation. The paper by Dudley and Matheson explore opinions from stakeholders on the Biomedical Scientist role. Questions were asked on how to recognise that Biomedical Scientists are meeting the HCPC standards and other professional guidelines to support the achievement of patient outcomes. Putting the patient at the centre scored highly as an essential aspect of the Biomedical Scientist role. Interestingly, a divergence of opinion was noted predominantly in the academic and student groups, thus identifying the possible existence of a gap between theory and practice. This research initiates the question that if such a gap exists, what strategies can be put into place to bridge this gap? Furthermore, how do higher education institutions ensure students graduate with the required skills and knowledge?

A recent article by Hussain and Hicks [8], assessed the employability skills of Biomedical Science graduates. The article highlighted perceived gaps in skills and knowledge by employers, which could negatively impact on the future workforce pipeline and subsequent service delivery. A subsequent study by Hussain et al. in this Special Issue explores the use of a practical session utilising an “Authentic Pathology Specimen Reception” within the biomedical science programme. The implementation this resource for developing biomedical science student competencies and employability demonstrates how simulation-based learning can be used as a tool to enhance the development of core biomedical science knowledge and strengthen employability of the graduate.

The paper by Garden describes how a collaboration with the Advanced Therapies Skills Training Network utilised current best practice to significantly impact upon teaching and workplace training in Scotland. The case provided insight on how to ensure biomedical sciences students graduate with the knowledge and skills required for employment within the Life Science sector.

Robertson et al. also describes an approach to enhancing graduate skills. This paper discusses the successful implementation of an assessment literacy strategy derived from a vocational veterinary teaching context and implemented as a foundational Biomedical Science learning activity. This concept highlights how teaching strategies can be affective across different disciplines and career pathways.

## Pedagogical Approaches and Student Attainment

Scenario-based learning and gamification have many advantages over traditional didactic lecture-style teaching methods [7]. May et al. explore the use of a scenario-based learning tool called Resimion, which had been adapted for Biomedical Science education. Resimion is a platform that blends applied learning pedagogy with gamification pedagogy. Learners work through problem- or scenario-based activities alone or collaboratory. Results of this study demonstrated good student engagement and positive feedback, with comparable results for neurodiverse and neurotypical groups.

Active learning pedagogy involves students in learning activities which promote doing, rather than listening [9]. Active learning is a tool which increases interactivity and stimulates engagement. Lees-Murdock et al. assess the efficacy of active learning in supporting student performance. They report how full engagement with an active learning approach, significantly correlates with increased student performance.

Student attainment and satisfaction was also explored in the study by Veuger et al. This study supporting students during their Biomedical Science UG Project Research project through a staff-student partnership. Students felt strongly that their experience, satisfaction and success was influenced by the student-supervisor relationship. This study and that by Millar et al. highlights the importance of staff student partnerships which ultimately promote student outcomes in relation to increased engagement, motivation, ownership and meta-cognitive learning.

The final paper in this Special Issue explores application of the theoretical principles of Malcolm Knowles’ theory of andragogy. Knapke et al. evaluated data collected from participants involved in science training workshops as part of a biomedical research setting. The paper collected data on the participants’ readiness to learn and problem-based learning orientation. Interestingly, the participants in this study were faculty, staff, and graduate students from the University of Cincinnati (UC). It would be interesting to see if their approach can be applied to Biomedical Science education and training in the United Kingdom and further afield.

## Future Aspirations Relating to Biomedical Science Education and Training

Although many current and evolving pedagogical concepts were explored in this Special Issue, future consideration is needed for the evolving nature of the digital age. In a generation where AI technologies are increasingly becoming available, educators and trainers need innovative ways to consider the pros and tackle the challenges associated with the development of robust, authentic and valid assessment. Furthermore, educators and trainers need to consider the preparedness of future biomedical scientists for an ever-changing pathology service, whether this involves automation, AI platforms or sustainable working. In the next Special Issue, it will be interesting to see how these aspects have been integrated into curriculums.

## Conclusion

This Special Issue presents a range of advances in theory, methodology and application of embedding workforce skills and knowledge requirements into current biomedical science education and development programmes. We hereby invite you to explore these articles in this Special Issue and consider applying these principles within to your own educational and training programmes.
